# Clinical trial and in-vitro study comparing the efficacy of treating bony lesions with allografts versus synthetic or highly-processed xenogeneic bone grafts

**DOI:** 10.1186/s12891-016-0930-1

**Published:** 2016-02-13

**Authors:** Eva Johanna Kubosch, Anke Bernstein, Laura Wolf, Tobias Fretwurst, Katja Nelson, Hagen Schmal

**Affiliations:** Department of Orthopedics and Trauma Surgery, Albert-Ludwigs University Medical Center, Freiburg, Germany; Department of Craniomaxillofacial Surgery, Albert-Ludwigs University Medical Center, Freiburg, Germany; Department of Orthopaedics and Traumatology, Odense University Hospital, Odense, Denmark; Department of Clinical Research, University of Southern Denmark, Odense, Denmark

**Keywords:** Bone substitute, Bone filler, Bone grafting, Allograft, Hydroxyapatite, BioOss, Clinical trial, Biomarker

## Abstract

**Background:**

Our study aim was to compare allogeneic cancellous bone (ACB) and synthetic or highly-processed xenogeneic bone substitutes (SBS) in the treatment of skeletal defects in orthopedic surgery.

**Methods:**

232 patients treated for bony lesions with ACB (*n* = 116) or SBS (*n* = 116) within a 10-year time period were included in this case–control study. Furthermore, both materials were seeded with human osteoblasts (hOB, *n* = 10) and analyzed by histology, for viability (AlamarBlue®) and protein expression activity (Luminex®).

**Results:**

The complication rate was 14.2 %, proportion of defects without bony healing 3.6 %; neither outcome parameter differed comparing the intervention groups. Failed consolidation correlated with an increase in complications (*p* < 0.03). The rate of complications was further highly significant in association with the location of use (*p* < 0.001), but did not depend on age, ASA risk classification, BMI, smoking behavior or type of insurance. However, those factors did significantly influence the bony healing rate (*p* < 0.02). Complication and consolidation rates were independent of gender and the filling substances employed within the different locations. Histological examination revealed similar bone structures, whereas cell remnants were apparent only in the allografts. Both materials were biocompatible in-vitro, and seeded with human osteoblasts. The cells remained vital over the 3-week culture period and produced microscopically typical bone matrix. We observed initially increased expression of osteocalcin, osteopontin, and osteoprotegerin as well as leptin and adiponectin secretion declining after 1 week, especially in the ACB group.

**Conclusion:**

Although both investigated materials appeared to be similarly suitable for the treatment of skeletal lesions in-vivo and in-vitro, outcome was decisively influenced by other factors such as the site of use or epidemiological parameters.

## Background

The treatment of large bone defects caused by trauma, degenerative or congenital diseases and tumor lesions is one of the greatest challenges in current orthopedic research, making the development of effective bone regeneration therapies a major topic. Although the amount of available autogenous cancellous bone is naturally limited, and graft harvesting from the iliac crest leads to significant donor-site morbidity [1], autogenous bone grafts combining osteogenic, osteoconductive and osteoinductive properties must be considered the gold standard for bone replacement for now [2]. The osseous regeneration capacity is limited in older patients especially, and donor site morbidity increases [3]. The known disadvantages of autologous bone grafting have prompted the search for alternatives. The long-term clinical goal is to regenerate an adequate amount of bony tissue with anatomical, physiological, three-dimensional morphology. In addition to bone applications, current bone regeneration strategies include cell-based or stem cell-based treatments, the application of bioactive factors such as BMP-2 and BMP-7, different biologic or artificial scaffolds and various combinations [2]. However, current and emerging therapies are still significantly restricted. Several artificial bone materials have recently been tested. Artificial bone grafts, most based on tricalcium phosphate (TCP) or hydroxyapatite (components of natural bone) usually only possess osteoconductive properties enabling adjacent osteoblasts to migrate [4]. Synthetic bone grafts are an alternative to autologous and allogeneic graft options, given their wide availability, comparatively low cost, slow biodegradation, superior strength in compression and the absence of risks such as donor-site morbidity and viral transmission. However, because they are strictly osteoconductive, their biologic role is limited, especially in fracture healing [5].

In the early 2000s more than 500,000 bone grafting procedures per year were carried out in the United States and 2.2 million worldwide in order to augment bone defects in orthopedics or other fields as dentistry [6]. Allogenous bone grafts are harvested from humans or human cadavers, undergo sterile processing and are transplanted to a recipient. Depending on the preparation process, allografts exhibit osteoconductive and sometimes osteoinductive potential. Allogeneic transplants can be infectious [7, 8], may trigger immunological reactions [9–11], and the sterilization process prior to transplantation leads to the loss of osteoinductive properties [12–14]. Recent studies by Ghanaati et al. [11] and Fretwurst et al. [15] revealed the presence of organic/cellular remnants in allografts and xenogeneic bone blocks commercially available for dental and orthopedic surgery that might induce an immune response. As freezing alone is insufficient in safety terms [16], terminal sterilization, usually irradiation, is carried out as well. However, the incorporation of allografts is reduced to approximately 40 % after irradiation [17, 18], compared to 80–100 % in non-irradiated grafts [19–21]. The freezing process also delays the vascularization of allografts after implantation [22], which might be detrimental, as vascularity has been identified as a central component influencing bone healing and necessary to effective graft repair [2]. The limited osteoinductive capacity of allografts presumably plays a fundamental role in allograft failure due to fracture or nonunion [14].

In contrast to adult hyaline cartilage, which does not restore its native structure once damaged, the human skeleton has a remarkable ability to regenerate after injury. Nevertheless, the conditions for spontaneous bone healing are not always ideal. Substantial bone loss in case of trauma or tumor lesions often requires bone-graft augmentation. Evidence of the effectiveness of graft incorporation and fracture healing remains elusive [23, 24]. Most of the literature addresses medium and long-term outcomes after revision arthroplasty [25]. In terms of bone grafting in fracture therapy, the latest data merely focus on certain anatomical locations [24, 26].

The aim of this study was to compare and determine the clinical effectiveness of allogeneic cancellous bone grafts (ACB) and synthetic or highly processed xenogeneic bone substitutes (SBS) for treating bony defects in different sites considering epidemiological and custom-designed parameters. Our rationale for comparing these two groups was the substantial differences described above, including the presumed presence of cell remnants in ACB in contrast to SBS. Nevertheless, we hypothesized that there would be no difference between ACB and SBS in their effectiveness in bone regeneration or consolidation. To clarify possible differences regarding biocompatibility and osteoconductivity, in-vitro testing was additionally carried out. Our hypothesis regarding the in-vitro experiments was that it is possible to predict in-vivo differences.

## Methods

The study is divided into a clinical and an experimental part.

### Patients

Within one decade, from 2001 to 2011, we identified 232 patients treated with ACB or SBS (117 female, 115 male; mean age 59 ± 18 years) based on the documented OPS code. The following SBS were used: BioOss® (Geistlich, Wolhusen, Switzerland) in 41.4 %, and at clearly lower percentages, respectively: Norian SRS® (Synthes) (8.6 %), Chronos® (Synthes) (1.7 %), Atlantik® (Argomedical) (2.6 %), Alaska® (Argomedical) (0.9 %), Endobone® (Biomet) (4.3 %), Pyrost® (Stryker) (0.9 %), Nanostim® (Medtronic) (37.1 %), Actifuse® (Baxter) (0.9 %), Tutobone® (Novomedics) (0.9 %) and PerOssal® (Botiss) (0.9 %). The SBS group thus contained various xenogenic or synthetic, commercially available bone substitutes, which, according to the manufacturers’ information, had undergone multiple purification processes and were supposed to be free of organic residues. Commercially-available human allografts, freeze-dried cancellous bone from femoral heads and freeze-dried cancellous cubes or blocks (DIZG, German Institute for Cell and Tissue Replacement, Berlin, Germany) were used in the ACB group. The study’s end points were defined as consolidation or treatment failure. We also determined the occurrence of complications. Similar to trials following the German Pharmaceuticals Act regulations, all complications were documented as adverse events. We investigated the relationship between a given complication and the use of a particular bone-graft material in both groups. When in doubt, we presumed a correlation between the complication and the use of bone graft, which was then documented as an adverse event. Basically, complications were defined as the need for surgical revision/re-operation or a patient’s death. Documented complications were: death, re-operation, infection/wound infection, seroma, pseudarthrosis, re-fracture, plate or screw fracture or loosening, secondary dislocation, loss of reduction, necrosis of the humeral head, cup/implant loosening, nerve palsy and cyst recurrence. We ultimately enrolled 116 patients treated with allografts and 116 patients treated with synthetic or highly-processed bone grafts in this study.

To ensure better comparability of the various bone-graft materials used, we defined influencing factors: besides gender and age, clinical outcome was assessed in regard to defect location, defect size, reason and type of application (fracture or bone defect for other reasons (e.g., bone cysts, osteochondritis dissecans, tumor); ACB or SBS), time to consolidation, duration of hospital stay, kind of insurance, BMI (body mass index), ASA (American Society of Anesthesiologists) risk classification, and smoking habits.

The study was approved by the Ethics Committee of the University of Freiburg (registration number 10010/15). All included individuals have declared their informed consent for the retrospective analysis of their treatment data.

### Experimental setup

To test their biocompatibility, the most frequently used representatives of ACB and SBS were seeded with human osteoblasts and cell viability and metabolic activity were analyzed. The cells’ preparation protocols were approved by the Ethics Committee of the University of Freiburg as part of the ‘Tissue bank for research in the field of tissue engineering’ project (GTE-2002) and the biobank ‘Osteo’ (AN-EK-FRBRG-135/14). Cancellous bone (DIZG, Berlin, Germany) and BioOss®-Granula (Geistlich, Wolhusen, Switzerland) in size of 3–4 mm were therefore used. BioOss® (Geistlich, Wolhusen, Switzerland) is a natural, nanocrystalline, carbonated hydroxyapatite of bovine origin. A patented multi-stage purification process prior to gamma-sterilization should remove proteins and de-activate viruses and other pathogens. The inorganic bone matrix with its macro- and microporous structure provides osteoconductive properties similar to human cancellous bone. We used BioOss® in further in-vitro experiments in the SBS group because of its frequent in-vivo application. ACB and BioOss® were examined for any cells or cellular remnants prior to colonization with hOB by histology.

### Histologic processing

Histologic processing was done as described [15]. The specimen was dehydrated in 100 % ethanol and subsequently infiltrated, embedded and polymerized in Technovit 9100 (Heraeus Kulzer, Wehrheim, Germany) according to the manufacturer’s instructions. After the polymerization process, samples were cut into 500 μm sections using a rotary diamond saw Secotom- 50 (Stuers, Ballerup, Denmark). The sections were mounted onto opac acrylic-slides (Maertin, Freiburg, Germany) and ground to a final thickness of approximately 60 μm on a rotating grinding plate (Stuers, Ballerup, Denmark). All specimens were stained with azure II and pararosaniline (Merck, Darmstadt, Germany).

Slides were imaged with an Axio Imager M1 microscope equipped with a digital AxioCam HRc (Carl Zeiss, Göttingen, Germany). The histologic sections were analyzed via analySIS FIVE – software (Soft Imaging System, Münster, Germany).

### Isolation of osteoblasts

Femoral heads were obtained during hip arthroplasty operations following femoral neck fractures. The material used was taken following informed consent from patients in accordance with the Ethics Committee of the University of Freiburg as part of the ‘Tissue bank for research in the field of tissue engineering’ project (GTE-2002) and the biobank ‘Osteo’ (AN-EK-FRBRG-135/14). The degree of osteoarthritis was evaluated on X-rays using Croft’s modification of the Kellgren and Lawrence grading system. Cells from patients with a Kellgren and Lawrence Score ≤2 were used in our experiments. Post-surgery human primary osteoblasts (hOB) were isolated within 8 h from cancellous bone by cell outgrowth from small pieces, as described [27]. Human OBs were cultured and expanded in medium 199 (Gibco, Carlsbad, CA), supplemented with 10 % FCS and 1 % penicillin-streptomycin at 37 °C and 5 % CO_2_. Human OBs from passage 1 or 2 were used in all further experiments.

### Cell seeding

Prior to application, we assessed cell viability, cytotoxicity and apoptosis events as well as specific osteogenic characteristics using ApoTox-Glo™ Triplex and AttoPhos® Assays (Promega, Madison, WI, USA) showing vital (>90 %) and typical osteoblasts. Similar amounts of BioOss® and allogeneic cancellous bone (20–25 mg) were weighed into 50 ml falcons and moistened with medium 199, respectively. Medium was discarded; bone grafts were seeded per falcon at a density of 1 × 10^6^ cells in 3 ml medium 199 and centrifuged at 1,200 rpm for 5 min. Cells were cultivated for 72 h at 37 °C and 5 % CO_2_ and then used in the experiments.

### AlamarBlue® - assay

The AlamarBlue®-Assay (AbD Serotec, Oxford, UK) is a quantitative test to measure the cell proliferation and viability of various human and animal cell lines, bacteria, plant and fungi. When cells are alive, they maintain a reducing environment within the cell’s cytosol. Resazurin, the active ingredient in AlamarBlue® reagent, is a non-toxic, cell permeable compound blue in color and highly fluorescent. Upon entering cells, resazurin is reduced to resorufin, a red compound that is highly fluorescent. Viable cells continuously convert resazurin to resorufin, increasing the overall fluorescence and color of the media surrounding cells [28].

Cells were cultivated for 1 week after isolation, followed by incubation with medium 199 containing 10 % AlamarBlue® for another 24 h. Cells were centrifuged for 5 min at 1,200 rpm, and supernatant collected to assess the resulting fluorescence. Cell-seeded constructs were washed with PBS, centrifuged and cultivated further in fresh medium. Supernatants were aliquoted into microtiter plates and the absorbance signal at 570 and 600 nm was detected on an ELISA plate reader. The same assay was repeated after 3 weeks’ cultivation.

### Milliplex® Human Bone Panel 1B

Milliplex® Human Bone Panel 1B Kit (Millipore AG, Zug, Switzerland) is a magnetic bead-based antibody microarray founded upon the sandwich immunoassay principle. The assay aims to quantify biomarkers that play a role in bone metabolism. We investigated the following analytes: osteocalcin (OC), osteopontin (OPN), osteoprotegerin (OPG), leptin and adiponectin. The assay was performed according to the manufacturers’ guidelines. Briefly, color-code microspheres are coated with a specific capture antibody. After a test sample’s analyte is captured by the bead, a biotinylated detection antibody is introduced and the reaction mixture is then incubated with streptavidin-PE conjugate, the reporter molecule, to complete the reaction on each microsphere’s surface. The Luminex®-100 reader is a system based on flow-cytometry principles. The reader identifies the bead’s color-code as well as the bead surface’s PE-fluorescence [29]. Analysis was performed with the following adjustments: 50 μl probe, 50 beads per set, gate 8,000–15,000, RP1 calibrator low PMT, time out 60 s. Protein was analyzed with the analytical software provided by the manufacturers.

### Scanning electron microscopy (SEM)

Scanning electron microscopy was performed after 1 and after 3 weeks of cultivation to observe the cell loading and growth patterns of hOBs on ACB and BioOss®. The cell-seeded grafts were centrifuged, supernatant discarded, and samples washed twice with PBS-buffer and fixed with fixation buffer overnight. Specimens were then dehydrated by rinsing them in an ascending ethanol-water mixture (30, 50, 70, 80, 90, 99 %; 2 × 5 min at RT for each step) and then rinsed in an ethanol-hexamethyldisalazin mixture (EtOH:HMDS 2:1, 1:1, 1:2, HMDS; 2x5 min at RT for each step). Finally, the samples were dried, sputtered with a thin layer of gold (SC7620 Mini Sputter Coater, Gala Instrumente, Bad Schwalbach, Germany), and examined under a Phenom scanning electron microscope (PHENOM Pure Desktop SEM, Phenom-World BV, Eindhoven, Netherlands).

### Statistical analysis

All values are expressed as mean ± standard deviation. Normality was tested by the Kolmogorov-Smirnov test. The paired *t*-Test was then used to assess differences among normally-distributed mean values. In case of heterogeneous variances, the Aspin-Welch Test was applied. Individual group mean scores were compared with the Mann–Whitney *U* test. The statistical comparison of incidences was done using the chi-square test. *p* < 0.05 was considered significant in all tests. Statistical analysis was carried out by the Statistical Package for the Social Sciences (SPSS) software version 21 (SPSS Inc., Chicago, USA).

## Results

### Characterization of patients treated with allografts

**Fig. 1:**
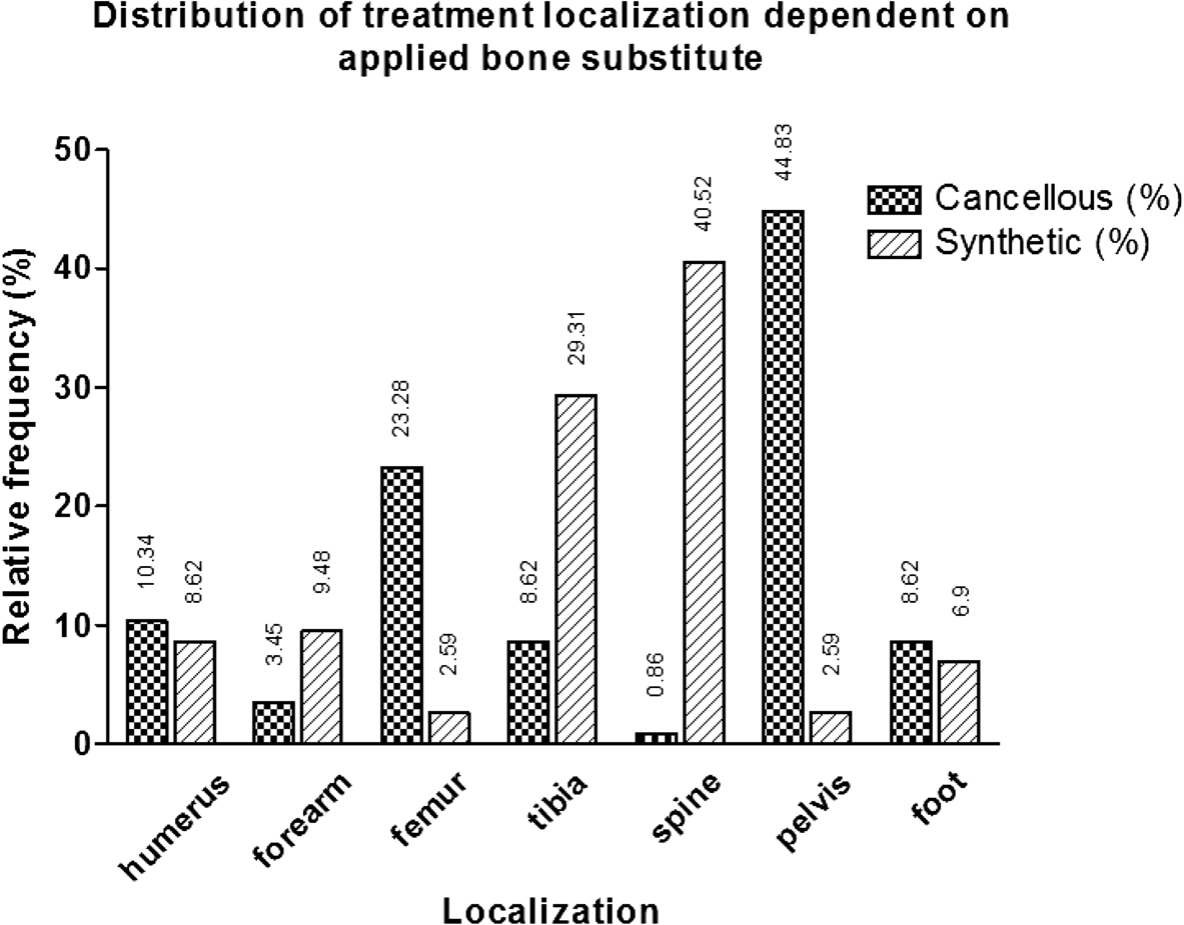
Distribution of treatment location according to the bone substitute; all cases in each group (allogeneic cancellous bone [ACB] or synthetic bone substitute [SBS]) amount to 100 %. Exact numbers are stated in the figure

116 patients were treated with ACB. The gender ratio was female/male (f/m) 62/54. The defect sites were distributed as: humerus 10 patients, forearm 4 patients, hip/pelvis 48 patients, femur 27 patients, tibia 10 patients, foot 10 patients, spine 1 patient (Fig. 1). 45 patients were treated for a fracture involving the loss of bone substance, 72 patients had a bony defect caused by other reasons such as tumor or cyst lesions requiring surgery. We observed bony consolidation of the defect in 104 patients, treatment failure in 6 patients, 6 patients were dropouts. The average time to consolidation was 3.93 ± 2.45 months; defect size averaged 20.21 ± 21.00 cm^2^. Mean age in this group was 62.6 ± 19.6 at the time of surgery, mean BMI was 26.01 ± 5.15 kg/m^2^. 23 patients were current smokers, the mean ASA risk classification was 2.31 ± 0.72 (Table 1). Complications occurred in 17 cases (14.7 %) (Table 2), 5 patients passed away during the follow-up period. Duration of hospital stay averaged 17.85 days. 90 patients had compulsory health insurance (C), 21 private health insurance (P) and 6 patients were covered by an employer’s mutual insurance association (E).

**Table 1:** Epidemiological and characterizing parameters

	Allogenic cancellous bone (ACB)	N	Synthetic bone substitutes (SBS)	N	P
Age	62.6 ± 19.6	116	55.8 ± 16.3	116	0.004^a^
Gender (f/m)	62/54	116	55/61	116	n.s.^b^
ASA	2.31 ± 0.72	116	1.95 ± 0.67	116	<0.0001^c^
Defect size	20.21 ± 21.00	114	5.75 ± 8.33	115	<0.0001^a^
BMI	26.01 ± 5.15	116	26.99 ± 5.60	116	n.s.^a^
Consolidation time (months)	3.93 ± 2.45	104	3.16 ± 3.31	107	n.s.^d^
Complication rate	17 (14.6 %)	116	16 (13.8 %)	116	n.s.^b^
Healing rate	104 (89.7 %)	116	107 (92.2 %)	116	n.s.^b^

Overview about the epidemiological and characterizing parameters comparing both intervention groups, *f* female, *m* male, *ASA* physical status according to the American Society of Anesthesiologists, *BMI* body mass index, *n.s.* – not significant, ^a^ *t*-Test, ^b^ *χ* ^2^-Test, ^c^ *U*-Test (Mann und Whitney), ^d^Aspin-Welch-Test

**Table 2:** Influence on complications

	Complication	N	No complication	N	
Age	63.52 ± 18.44	33	58.47 ± 18.24	199	n.s.^a^
Gender (f/m)	20/13	33	97/102	199	n.s.^b^
ASA	2.24 ± 0.66	33	2.11 ± 0.72	199	n.s.^c^
Defect size	10.98 ± 14.48	33	13.28 ± 17.95	196	n.s.^a^
BMI	26.79 ± 5.41	33	26.45 ± 5.39	199	n.s.^a^
Cancellous/Synthetic bone	17/16	33	100/99	199	n.s.^b^
Smoker/Non-Smoker	10/23	33	49/150	199	n.s.^b^
Insurance status W/C/P	23.5 %/13.4 %/12.2 %	32	76.5 %/86.6 %/87.8 %	198	n.s.^b^
Healing rate	7 (21.2 %)	33	1 (0.5 %)	186	<0.0001^b^
Consolidation time (months)	4.79 ± 4.39	26	3.36 ± 2.64	185	0.02^a^

Overview about the comparing parameters in patients with or without a complication, *f*female, *m* male, *ASA* physical status according to the American Society of Anesthesiologists, *BMI* body mass index, *n.s.* not significant. *W* employers mutual insurance association, *C* compulsory health insurance fund, *P* private health insurance fund. ^a^ *t*-Test, ^b^ *χ* ^2^-Test, ^c^ *U*-Test (Mann und Whitney)

### Characterization of patients treated with synthetic and highly processed bone grafts

116 patients were treated with SBS. The f/m gender ratio was 55/61. 41.4 % of the patients (*n* = 48) were treated with BioOss® (Geistlich, Wolhusen, Switzerland). The defect sites were distributed as: humerus 9 patients, forearm 10 patients, pelvis 3 patients, femur 3 patients, tibia 32 patients, foot 8 patients, spine 44 patients (Fig. 1). Bony consolidation was observed in 107 patients, treatment failure in 2 patients and the healing state in 7 patients remained unknown. The average time to consolidation was 3.16 ± 3.31 months; defect size averaged 5.75 ± 8.33 cm^2^. 52 patients were treated for a fracture, 64 patients were suffering from bony defects for other reasons. This group’s mean age at the time of surgery in was 55.8 ± 16.3 years, mean BMI was 26.99 ± 5.60 kg/m^2^. 36 patients were current smokers, the mean ASA risk classification was 1.95 ± 0.67 (Table 1). Complications occurred in 16 (13.8 %) cases (Table 2), 1 patient passed away during follow-up. Mean duration of hospital stay was 14.22 days. 82 patients had compulsory health insurance (C), 19 private health insurance (P) and 11 patients were covered by an employer’s mutual insurance association (E). To summarize: both groups (4.1. and 4.2.) differed substantially in age, co-morbidities and defect size, as indicated in Table 1.

### Clinical results

**Fig. 2:**
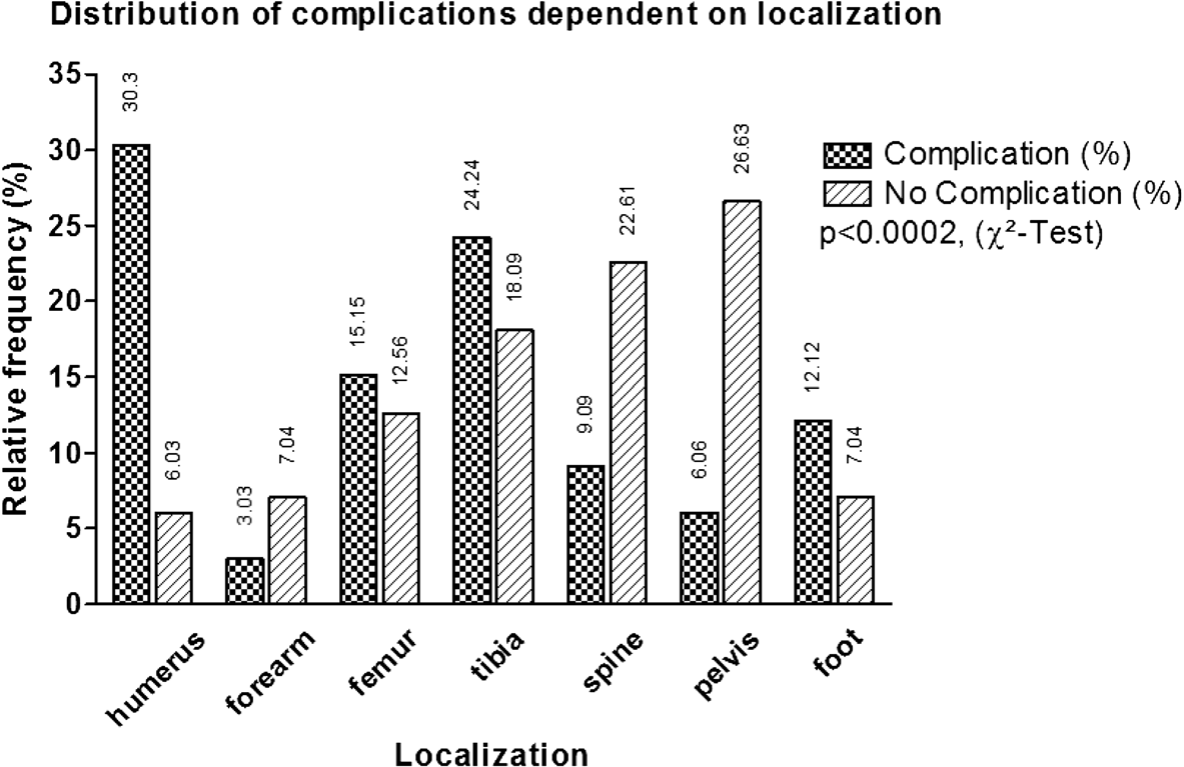
Frequency of complications according to the location where the various bone substitutes were applied; all patients in each group (complication or no complication) amount to 100 %. Exact numbers are stated in the figure

The complication rate reached overall 14.2 %; Table 2 compares the examined parameters in the groups with or without complication. The proportion of defects without bony healing was 3.6 %; Table 3 compares the examined parameters in the groups with or without healing. Failed consolidation correlated with an increase in complications (*p* < 0.03). The complication rate correlated very closely with the site of use (*p* < 0.0002) (Fig. 2), but not with age, gender, ASA risk classification, BMI, smoking behavior or type of insurance (Table 2). However, these factors significantly influenced the bony healing rate (*p* < 0.02) (Table 3). Complication and consolidation rates were independent of gender and the filling substances applied within the different sites. We detected no significant differences in the effectiveness of treating bony lesions with allografts versus synthetic or highly-processed xenogeneic bone grafts in various sites (Table 4). Higher complication rates were documented in association with the treatment of bony lesions in the humerus, tibia and the foot (Fig. 2). In the vicinity of the humerus we documented screw perforation, secondary loss of reduction, wound infection and osteonecrosis of the humeral head. Complications in the treatment of bony lesions of the proximal tibia were mainly secondary loss of fracture reduction, deep infections and wound infections. Wound infections were the main complication that occurred in the foot area. Considering the SBS group’s heterogeneity, further stratification was analyzed comparing complication and consolidation rates by discriminating between synthetic and highly-processed xenogeneic materials within the SBS-group in addition to the allografts (ACB) 116/61/55). Again, we found no statistically significant differences (p for chi square test >0.05 for all comparisons) in any endpoint (complications: ACB 17/99, synthetic 7/54, xenogeneic 9/46, consolidation failure: ACB 6/105, synthetic 0/58, xenogeneic 2/48, missing values 13).

**Table 3:** Influences on bone healing

	Bony healing	N	No healing	N	P
Age	57.89 ± 18.01	211	72.51 ± 13.45	8	0.0025^a^
Gender (f/m)	102/109	211	6/2	8	n.s.^b^
ASA	2.09 ± 0.71	211	2.75 ± 0.46	8	0.014^c^
Defect size	12.90 ± 17.62	211	18.51 ± 20.54	8	n.s.^a^
BMI	26.42 ± 5.38	211	26.94 ± 4.19	8	n.s.^a^
Cancellous/Synthetic bone	104/107	211	6/2	8	n.s.^b^
Smoker/Non-Smoker	51/160	211	5/3	8	0.014^b^
Insurance status W/C/P	13/159/38	210	3/3/1	7	0.0012^b^

Overview about the comparing parameters in patients with or without bony consolidation, *f*female, *m* male, *ASA* physical status according to the American Society of Anesthesiologists, *BMI* body mass index, *n.s.* not significant, *W* employers mutual insurance association, *C* compulsory health insurance fund, *P* private health insurance fund. ^a^ *t*-Test, ^b^ *χ* ^2^-Test, ^c^ *U*-Test (Mann und Whitney)

**Table 4:** Bone healing and complications within different locations

	Consolidation yes/no		N	P	Complication yes/no		N	P
	Cancellous	Synthetic			Cancellous	Synthetic		
Humerus	10/0	8/1	19	n.s.^a^	5/7	5/5	22	n.s.^a^
Forearm	4/0	10/0	14	n.s.^a^	0/4	1/10	15	n.s.^a^
Femur	24/3	3/0	30	n.s.^a^	5/22	0/3	30	n.s.^a^
Tibia	9/1	31/1	42	n.s.^a^	3/7	5/29	44	n.s.^a^
Spine	0/1	44/0	45	n.s.^a^	1/0	2/45	48	n.s.^a^
Pelvis	47/1	3/0	51	n.s.^a^	2/50	0/3	55	n.s.^a^
Foot	10/0	8/0	18	n.s.^a^	1/9	3/5	18	n.s.^a^

Number of complications and consolidation rates dependent on the group of applied bone substitutes, *n.s*. not significant, ^a^ *χ* ^2^-Test

### In-vitro results

#### Histologic findings

**Fig. 3:**
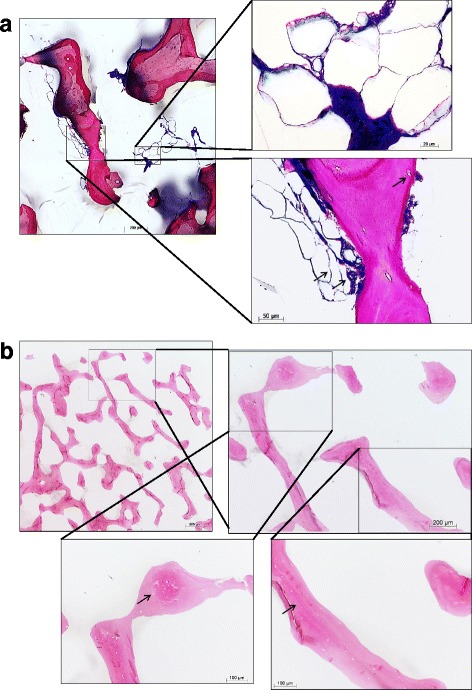
**a/b**: Histologic overview of allogeneic human cancellous bone (cryopreserved, caput femoris, DIZG) prior to use (**a**). Osteocytes and adipocytes are visible (arrows). Newly-formed bone is stained dark magenta, older bone light magenta. Soft tissue (blue) with fibrocytes (arrow) is visible adjacent to the bone trabeculae. Undecalcified ground sections, stained with azure II and pararosaniline, original magnification x 100 and x 400. The highly-processed xenogeneic bone graft (Orthoss®/BioOss®) prior to use does not exhibit any cells or cell remnants (**b**). In contrast to allogeneic cancellous bone, no soft tissue (blue) is detectable. Empty osteocyte lacunae are visible (arrows). Undecalcified ground sections, stained with azure II and pararosaniline, original magnification x 50, x 100 and x 200

Osteocytes, adipocytes and fibrocytes were detected in ACB (Fig. 3a). No cells or cellular remnants were detectable on BioOss® prior to cell seeding with hOB. Osteocyte lacunae were empty (Fig. 3b, arrows). In contrast to ACB, no soft tissue was detected.

#### Alamar Blue®- assay

**Fig. 4:**
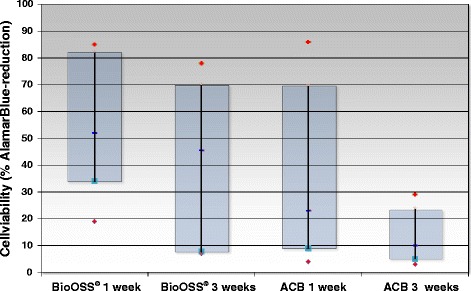
Cell viability of hOB cultured on either ACB or BioOss® was measured using the AlamarBlue® assay. Cells were vital during the 3-week culture period independent of the biomaterial used. No significant differences appeared between the two intervention groups and various points concerning cell viability and proliferation

Results were analyzed by plotting absorbance versus compound concentration, and we calculated the absolute reduction. This value correlates with cell number and metabolic activity. Human osteoblasts cultivated on BioOss® revealed a reduction in resazurin after 1 week in the AlamarBlue®-assay (53.1 ± 25.1 %); 41.8 ± 29.45 % after 3 weeks. Osteoblasts cultivated on cancellous bone (ACB) reduced resazurin by 35.2 ± 32.1 % after 1 week, and 13.3 ± 9.8 % after 3 weeks of total resazurin applied. Although the absolute resazurin reduction (cell number and metabolic activity) decreased over time especially in the allograft group (ACB), we detected no statistically significant differences between the two intervention groups and the two time points of cultivation (Fig. 4).

#### Milliplex Human Bone Panel 1B

We identified and quantified bone-specific biomarkers in the supernatant after 1 and 3 weeks of cultivation. Osteocalcin (OC), osteopontin (OPN), osteoprotegerin (OPG), leptin and adiponectin were detected and analyzed, revealing maintenance of the cells’ stable osteogenic phenotype independent of the biomaterial employed.

**Fig. 5:**
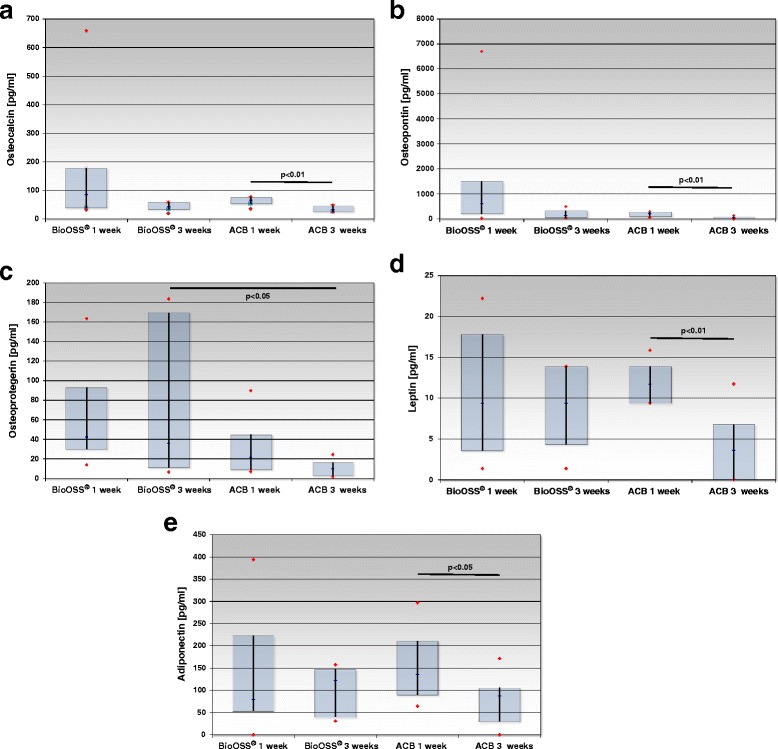
**a** Osteocalcin (OC) was detected in all probes. Although we noted a tendency toward slightly better results in the BioOss®-group and after 1 week’s cultivation; differences were statistically significant only in the ACB group (allogeneic cancellous bone), decreasing from 1 to 3 weeks (*p* = 0.002). **b** The same pattern is observed for osteopontin (OPN), which was detectable in all samples but revealed a tendency toward slightly better results in the BioOss®-group and after 1 week of cultivation; differences were statistically significant in the ACB (allogeneic cancellous bone) group only, decreasing from 1 to 3 weeks (*p* = 0.0045). **c** Osteoprotegerin (OPG) was also secreted by human osteoblasts seeded onto BioOss® or allograft (ACB). Here again the tendency toward slightly better results in the first 1 week: OPG concentrations in the supernatant of cultivated grafts were higher in the BioOss® group after 3 weeks (*p* = 0.039). **d** Leptin was detected in all probes. Comparing the different biomaterials and time points: differences attained statistical significance in the ACB group only (allogeneic cancellous bone), decreasing from 1 to 3 weeks (*p* = 0.002). **e** Adiponectin was detected in all probes. Comparing the different biomaterials and time points: differences attained statistical significance in the ACB group only (allogeneic cancellous bone), decreasing from 1 to 3 weeks (*p* = 0.04)

Osteocalcin, a marker of bone anabolism, was detected in all probes. Mean OC concentrations in cell culture supernatants of human osteoblasts (hOB) cultivated on BioOss® for 1 week were 160.2 ± 85.2 pg/ml; 62.9 ± 4.6 pg/ml in the group of cancellous bone. Concentrations fell after 3 weeks in both groups to 43.7 ± 6.1 pg/ml (BioOss®) and 34.5 ± 3.7 pg/ml (ACB), respectively. The ACB group’s concentrations decreased significantly from 1 to 3 weeks (*p* = 0.002) (Fig. 5a, but there was no statistically significant difference between the groups analyzing the respective time points.

Mean concentrations of osteopontin were 1,514.2 ± 883.9 pg/ml after 1 week in supernatants of osteoblasts cultivated on BioOss®. Concentrations decreased after 3 weeks to 187.5 ± 70.1 pg/ml. Osteoblasts cultivated on cancellous bone (ACB) showed mean concentrations of 185.2 ± 32.1 pg/ml after 1 week and 51.6 ± 14.8 pg/ml after 3 weeks of cultivation. The ACB group’s OPN concentrations dropped significantly from 1 week to 3 weeks (*p* = 0.0045). OPN concentrations were lower on cancellous bone after 1 (*p* = 0.050, n.s.) and after 3 weeks compared to concentrations on BioOss® (*p* = 0.052, n.s.) without reaching statistical significance (Fig. 5b).

Osteoprotegerin was secreted by human osteoblasts seeded onto BioOss®; concentrations averaged 61.7 ± 19.4 pg/ml after 1 week and 73.3 ± 32.5 pg/ml after 3 weeks. Osteoblasts seeded onto cancellous bone displayed concentrations of 29.7 ± 9.0 pg/ml after 1 week and of 10.6 ± 2.8 pg/ml after 3 weeks. OPG was expressed less in the supernatant of osteoblasts seeded onto cancellous bone when compared to BioOss® after 1 (*p* = 0.081, n.s.) and after 3 weeks (*p* = 0.039) (Fig. 5c).

We detected leptin after 1 week in the BioOss® group at concentrations of 10.7 ± 2.9 pg/ml, after 3 weeks of 8.9 ± 1.99 pg/ml; concentrations of 11.9 ± 0.9 pg/ml and 4.1 ± 1.5 pg/ml were measured in the ACB group, respectively. The ACB group’s decrease was significant from 1 to 3 weeks (*p* = 0.002) (Fig. 5d), but there was no statistically significant difference between the groups analyzing the respective time points.

Mean adiponectin concentration in the BioOss® group after 1 week reached 130.3 ± 50.9 pg/ml, and 103.1 ± 21.9 pg/ml after 3 weeks. Mean concentration in the allograft group after 1 week was 153.0 ± 25.3 pg/ml, and after 3 weeks 76.9 ± 19.2 pg/ml. The ACB group’s decrease from 1 to 3 weeks was again significant (*p* = 0.04) (Fig. 5e), but there was no statistically significant difference between the groups analyzing the respective time points.

Whichever graft material was used, human osteoblasts seeded onto ABC or SBS maintained their biochemical characteristics. In short: the expression of osteogenic biomarkers of human OB cultured on different graft materials is similar and reveals a time-dependent regulation pattern in-vitro.

### Scanning electron microscopy (SEM)

**Fig. 6:**
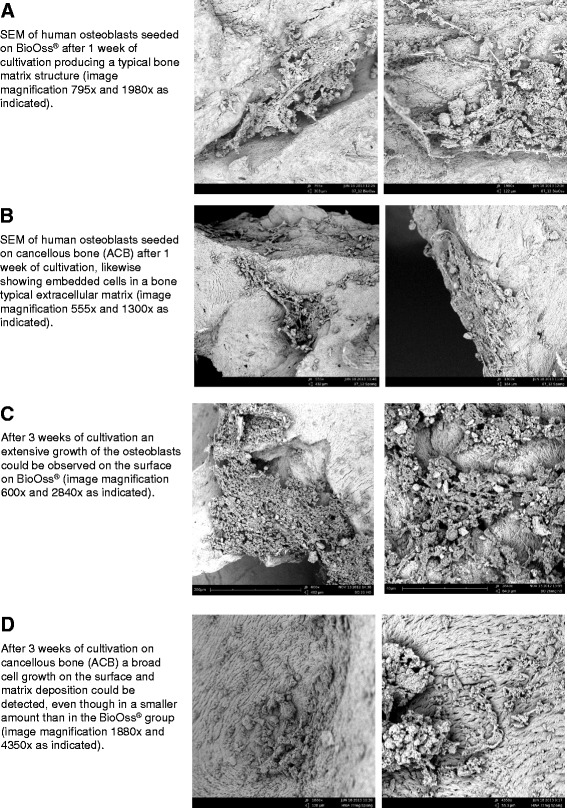
**a** SEM of human osteoblasts seeded on BioOss® after 1 week’s cultivation producing a typical bone matrix structure (image magnification 795x and 1980x as indicated). **b** SEM of human osteoblasts seeded on cancellous bone (ACB) after 1 week’s cultivation, likewise showing embedded cells in bone-typical extracellular matrix (image magnification 555x and 1300x as indicated). **c** After 3 weeks of cultivation, extensive osteoblast growth is visible on the BioOss®-surface (image magnification 600x and 2840x as indicated). **d** After 3 weeks of cultivation on cancellous bone (ACB), broad cell growth on the surface and matrix deposition are visible, although to a lesser extent than in the BioOss® group (image magnification 1880x and 4350x as indicated)

Electron microscopy showed a homogeneous distribution pattern of osteoblasts seeded onto different bone graft materials, good adhesion to the material and the pericellular deposition of extracellular matrix. We also noted that that after just one week, osseous matrix adhered to the implants in both groups (Fig. 6a/b). After 3 weeks, intimate contact with osteoblasts embedded in a mineralized, fibril-rich extracellular matrix was present on both ACB and SBS (BioOss®). Although there were no significant differences between the two groups in cell viability or proliferation, semiquantative impression indicated slightly more extensive osteoblast growth on the BioOss® surface (Fig. 6c/d). We observed no interpositioned interfacial layer or foreign-body reactions (not expected) in any of the samples.

## Discussion

Aim of this study was to compare the effectiveness of treating bony lesions with allografts (ACB) versus synthetic or highly-processed xenogeneic bone grafts (SBS) in terms of patient-dependent factors and the various sites of the bone defects. The key finding we made in this study is the similar efficacy of ACB and SBS in the treatment of skeletal lesions in-vivo and in-vitro, confirming our hypothesis. Other factors such as the location of use and epidemiological aspects have a greater impact on the clinical outcome than does the type of bone graft.

We detected no significant differences between the ACB and SBS groups either in their clinical findings or in-vitro biocompatibility. The consolidation rate was influenced significantly by epidemiological and patient-dependent factors such as age, ASA risk classification, smoking habits and type of insurance, but not by the bone-graft material used. Interestingly, although the defect size differed significantly between the groups, the consolidation rate was not influenced by it. The complication rate in our study correlated very significantly with defect location. Complication and consolidation rates did not display significant differences between ACB and SBS within the same location, however. Furthermore, the complication rate was also not influenced significantly by the biomaterial group used, but the bony-healing rate did correlate significantly with the type of insurance, revealing worse outcomes in patients covered by an employer’s or compulsory health insurance in comparison to those with private coverage. While patients cannot influence the injury site and/or fracture or defect characteristics, bony healing may be influenced by epidemiological and patient-dependent factors, such as a healthy life style. Well-educated patients are more likely to have private health insurance and show potentially better compliance, factors that would explain these clinical findings.

Current evidence-based treatment paradigms for bone grafting in bony defects or fracture healing remain still elusive and are frequently controversial [23, 24]. Most of the available literature addresses medium- and long-term outcomes after revision arthroplasty [25]. The latest data on bone grafting in fracture care merely focuses on certain anatomical locations [24, 26]. There is very little data on bone substitutes and a paucity of randomized controlled studies in particular, an observation also supported by a Cochrane review from Board et al. concerning the comparison of processed versus fresh-frozen bone for impaction bone grafting in revision hip arthroplasty. No randomized controlled trial comparing the clinical use of processed versus fresh-frozen bone in revision hip surgery has been published. Surgeons’ choices of bone graft nowadays are more apt to be based on personal preference and the graft material’s availability than on evidence [23]. Our data do not contradict to this approach, because we could show that complication rates after bone grafting of the hip is associated with a high success rate independent on the used material. In contrast, several randomized trials on bone-graft alternatives for spinal fusion procedures have been published. Approximately 1,400 products for use as bone void fillers are available on the international market [30]. Most of the trials compare the clinical effectiveness of allografts, synthetic/processed bone grafts or ceramics with autologous bone grafting. Thalgott et al. conducted a prospective, blind, single-site study to evaluate the outcomes and fusion rates of anterior lumbar interbody fusions with fresh-frozen or freeze-dried femoral ring allograft as part of a circumferential fusion in 40 patients. Radiography revealed fusion in 71.4 % of the 40 fusion levels [31]. Correlating with our data, smoking was in this study strongly associated with pseudarthrosis development. 58.3 % of the smokers who received freeze-dried allografts and 16.7 % of the smokers who underwent fresh-frozen allografts required revision for non-unions. In contrast, only one non-smoker (7.1 %) required revision for pseudarthrosis after a freeze-dried allograft and another non-smoker after a fresh-frozen allograft. Those results support our study findings and confirm the influence of smoking on the consolidation rate. Suchomel et al. conducted a prospective semi-randomized study comparing allograft to iliac crest bone graft in 79 patients undergoing one- or two-level anterior cervical discectomy and fusion, detecting no difference in graft migration or collapse. They noted a significantly slower fusion rate at 3 and 6 months in the allograft group, which was no longer present at 1 and 2 years [32]. A possible explanation are the immunological consequences of the histologically shown cell remnants that were found during our investigation. Several other studies confirm good consolidation rates in the field of spine fusion independent of the graft material [30, 33], which would be in line with our finding that this anatomical location provides a good biological environment for graft integration. In their prospective, randomized study of 29 patients, McConnell et al. observed 100 % fusion rates in anterior cervical discectomy and fusion with either coralline hydroxyapatite or iliac crest bone grafting. However, the hydroxyapatite group displayed a significantly higher rate of graft fragmentation and settling [30, 34]. Fischer et al. recommend the use of ceramics in common spinal fusions as osteoconductors, but not without employing additional osteoinductive material [30]. In fact, a combination of autologous graft material and SBS was used in 44 out of 45 cases of bone grafting in the spinal area in our study. Overall, good consolidation rates in the field of spine fusion may result from the combination of the osteoconductive properties of SBS and osteoinductive capacities of autologous graft material. Furthermore, a usually ample blood supply may support consolidation in spine fusion independent of different osteoconductive graft materials. Nevertheless, problems arise with the use of different graft materials when consolidation is radiologically assessed. A scintigraphic study of 12 patients comparing hydroxyapatite grafts and iliac crest bone graft showed the same rate of uptake until fusion. Therefore, the authors concluded that the radiolucent line is not always a sign of pseudarthrosis in patients treated with hydroxyapatite [35]. As long as the fusion confirmation is based on X-ray or CT-scan, it is hard to differentiate between the bone graft material itself and a lack of bone ingrowth. Multiple studies on clinical outcomes after posterolateral lumbar fusion using ceramics as bone graft extender have reported good results. Hence, Fischer et al. hypothesize that the radiographic findings represent new bone formation [30].

The challenges of post-interventional radiologic assessment lead to the subject of bone grafting in fracture care. Unlike autograft or allograft, the total radiographic disappearance of hydroxyapatite is not to be expected, since its incorporation is associated with osseous ingrowth and slight marginal resorption. Hydroxyapatite possesses osteoconductive properties and serves as a scaffold, in contrast to the complete remodeling that occurs with auto- and allografts. Concerning bone grafting in fracture care, current data basically focuses on certain anatomical locations [24, 26]. Herrera et al. reported good clinical results in 17 patients after external fixation and cancellous allografting with freeze-dried, irradiated cancellous bone in unstable distal radius fractures. In all cases, bone-graft incorporation was evident, non-union did not occur. Incorporation was defined as the loss of allograft radiodensity and trabeculae crossing the fracture line [26]. A study by Arora et al. evaluated complications following internal fixation using a palmar 2.4 mm-locking compression plate in unstable distal radius fractures, with most complications involving tendon irritation. Delayed fracture union occurred in 3 patients, and 1 patient experienced intra-operative intra-articular screw displacement [36]. Correlating with the described excellent bone healing capacity of the forearm, we detected no non-union after bone grafting in the forearm in our study, and only one complication. The risk of non-union is minimal in distal radius fractures. Consequently, bone-graft substitutes are primarily used to provide structural stability and fill larger bony defects [37]. Furthermore, advances in plate design and technology such as locking plates [38] reduce the field of application for bone grafts in distal radius fractures. There is still a lack of robust evidence concerning bone grafting in distal radius fractures. A Cochrane Database analysis of randomized clinical trials concluded that there was insufficient evidence regarding functional outcomes and safety in the use of bone grafts and substitutes to treat distal radius fractures [39]. Bone quality, the size of the bony defect, blood flow to the fracture site, and the method of fixation/immobilization, as well as patient-dependent epidemiological factors all affect the healing process and maintenance of fracture reduction. As shown by our data, different anatomic locations entail varying levels of bone-forming activity and stability. Therefore, a single study validating the use of a bone-graft material in one location may not predict its performance in another anatomic site. Good clinical results concerning bone grafting in spine surgery, hip-joint arthroplasty or its application in distal radius fractures [25, 30, 39, 40] are in line with our results from both groups of bone-graft materials in those locations.

With tibial plateau fractures, restoration of the plateau surface is a key treatment factor. Residual depression zones in the weight-bearing area on the tibial plateau bear the risk of axis deviation and posttraumatic osteoarthritis [41]. However, restoring the tibial-plateau anatomy often leaves larger cancellous bone defects, especially when bone fragments have become impacted within the soft metaphyseal bone. Various bone-graft materials are available and have been used to fill metaphyseal cancellous bone defects. Various challenges exist, such as inferior mechanical properties, donor-site morbidity in autografting, toxic and exothermic reactions when using traditional polymethyl-methacrylate cement, graft-material dislocation and custom-designed challenges such as graft preshaping or the inappropriate application form. Lobenhoffer et al. conducted a prospective study of 26 patients suffering from type B2, B3 and C3 fractures of the tibial plateau using an injectable calcium phosphate bone cement. 2 patients presented partial secondary loss of reduction, one of whom could not comply with partial weight bearing [42]. Veitch et al. presented a study of 6 patients treated with compaction morselized fresh-frozen bone allograft in tibial-plateau fractures. Despite their small cohort, the authors recommend the treatment of tibial-plateau fractures with fresh-frozen allograft because of its good graft incorporation, good remodeling and lower costs [24]. Several single-site studies addressing the use of different bone-graft materials in tibial-plateau fractures are available, but there is little data from controlled, randomized studies of bone grafting in tibia-plateau fractures. A current study protocol of Nusselt et al. is comparing the treatment of fracture defects in tibial-plateau fractures with a bioresorbable hydroxyapatite/calcium sulphate cement (Cerament™ Bone Void Filler) and autologous bone grafting [43]. Correlating with the comparable experiences of different material classes, we observed no significant group differences in consolidation or complication rates in tibial-plateau fractures in our study. 76 % of our study patients with a tibial-plafond fracture underwent fracture treatment with SBS to prevent the donor-site morbidity associated with autografting and potential immune reactions after allografting for relatively smaller bone defects. Only 2 failures of consolidation were documented, whereas an 18 % complication rate in tibial-plateau fracture therapy in both intervention groups was recorded. The complications we observed are in line with the current literature concerning tibial-plateau fractures, as they tend to entail secondary loss of reduction and wound infections [24, 44].

There is a paucity of evidence-based data on bone grafting in humeral bone defects. Daugaard et al. conducted a study in 20 canine proximal humerus, examining the combined effect of parathyroid hormone and bone graft on implant fixation [45]. Taehoon et al. evaluated the effect of β-tricalcium phosphate and poly-L-lactide-co-glycolide-co-epsiolon-caprolactone membrane in canine-humerus defects, obtaining the expected results from the osteoconductive material, leading to the original morphology’s restoration. However, the new cortical bone was thinner and less well organized than the adjacent intact cortex, and the amount of new cancellous bone was also sparse [46]. Studies in humans mainly focus on fracture care, the anatomical characteristics of different fracture locations, and frequent complications [47–50]. Südkamp et al. conducted a prospective, multicenter, observation study investigating the open reduction and internal fixation of proximal humeral fractures employing a proximal humerus locking plate; 34 % complications were registered in 155 patients. 40 % of the complications were attributable to problems of surgical technique, mostly the intra-operative perforation of the humeral head by a screw. Further complications included plate fracture (1.9 %), impingement (2.6 %), pseudarthrosis (2.6 %), wound infection (3.9 %), loss of reduction (7.1 %), and necrosis of the humeral head (3.9 %) [51]. Although the general complication rate in our study cohort amounted to only 14.2 %, the location-specific complication rate was 30.3 % for the humerus. This higher proportion is in line with the findings of Südkamp et al. and seen after fracture care of the proximal humerus with a locking plate. A failure of consolidation in the humerus only occurred in 1 of 19 patients (SBS), whereas 10 complications occurred (5 in each group). Good consolidation rates in both groups indicate that treatment efficacy depends on other influencing factors (such as epidemiological aspects, as well as local factors such as blood supply, bone quality, anatomical characteristics and mechanical demands) rather than on the bone-graft material, at least when it merely provides osteoconductive properties. Other bone-graft techniques in humeral fractures aim to prevent known difficulties. Khmelnitskaya et al. described a D.G. Lorich technique to treat 4-part proximal humerus fractures using an intramedullary fibula strut graft as a reduction aid and as a structural augmentation for screw purchase in patients with poor bone quality. This technique seems to avoid frequent problems in humeral-fracture care such as screw penetration or hardware cut-out [47], but it does not improve the already good consolidation rate.

BioOss® was most frequently used in the SBS group, thus we decided to compare the in-vitro characteristics of ACB and BioOss®. Allografts and highly-processed bovine hydroxyapatite showed similar in-vitro osteoconductive properties and similar biocompatibility. Human osteoblasts on both materials maintained their stable osteogenic phenotype, resulting in the expression of similar amounts of osseous marker proteins. SEM imaging demonstrated good adherence of the osteoblasts on both materials. After a 3-week culture, osteoblasts were organized as nodular aggregations, and the amount of deposited extracellular matrix was greater than that after 1 week’s cultivation. SEM imaging demonstrated dense collagen nanofibers deposited on the scaffold spread homogenously and covering the entire surface. Those findings concur with the results of Sachar et al., who investigated osteoblasts gained from mice calvaria on three-dimensional nanofibrous gelatin scaffolds [52]. However, they noted an increase in protein synthesis and greater cell density in 5-day cultures compared to cultures lasting 14 days. We hypothesized that the decrease in protein and hormone concentrations in both our study groups may be caused by a limited amount of medium substrates in the in-vitro cultures. Another reason for the decreasing proliferation and protein synthesis in-vitro in our study might be the use of human osteoblasts isolated from elderly patients who had undergone hip-arthroplasty surgery following femoral neck fractures, whereas many other studies employed murine osteoblasts [52, 53].

We examined the protein and hormone synthesis of OPN, OC, OPG, leptin and adiponectin in osteoblasts on ACB and SBS. Osteopontin, a glycoprotein also known as bone sialoprotein I, is a linking protein that binds hydroxyapatite, and its cell-adhesion properties are required for osteoclastogenesis. OPN is expressed during the early stages of differentiation of osteoclast and osteoblast progenitors. OPN and OC are considered the most sensitive markers for bone-specific tissue formation [54, 55]. After 1 week of cultivation on BioOss®, we detected high levels of OPN and OC, whereas the early marker OPN decreased especially after 3 weeks. OC levels on ACB fell significantly after 3 weeks, indicating a lack of substrates or a lower proportion of matrix-embedded cells on ACB. Furthermore, diminished protein synthesis in osteoblasts isolated from elderly patients is conceivable. OPG and RANKL are known to be essential effectors for osteoclastogenesis [56–58]. OPG, a cytokine receptor and member of the tumor necrosis factor receptor superfamily secreted by osteoblasts, inhibits differentiation of the osteoclast precursor into mature osteoclasts by attaching to RANKL and inhibiting osteoclastogenesis [59]. An increasing level of OPG on BioOss® from 1 to 3 weeks indicates an anabolic condition, whereas OPG on cancellous bone was expressed significantly less after 3 weeks. Luo et al. described the interrelation of adiponectin and the RANKL/OPG axis on bone metabolism. Adiponectin induces RANKL and inhibits OPG expression in human osteoblasts through the AdipoR1/p38 MAPK pathway, inducing osteoclast formation [60]. The different dynamics in protein levels of adiponectin and OPG on BioOss®, together with a rise in OPG after 3 weeks and a falling level of adiponectin therefore reveal anabolic bone metabolism on BioOss® after 3 weeks. The adiponectin level on ACB rises, whereas OPG decreases, indicating a catabolic condition, which may be supported by overall lower protein levels (OC, OPN) on ACB in comparison to BioOss®. The role of leptin, a pluripotent hormone linked to human body fat regulation in bone metabolism is controversial [61–63]. Reseland et al. demonstrated leptin secretion of human osteoblasts, which promotes osteoblastic cell growth and bone mineralization via an autocrine and endocrine mechanism [64]. However, the expression of leptin seems to be restricted to the mineralization and/or osteocyte transition period during hOB differentiation [65]. The nearly-constant leptin level of hOB cultured on BioOss® from 1 to 3 weeks indicates the ongoing mineralization phase, which is supported by SEM findings showing ample matrix deposition, whereas a significant decrease was observed in the leptin level on ACB from 1 to 3 weeks. Significant differences between the protein and hormone synthesis on ACB and SBS were only detected in conjunction with particular parameters in a temporal pattern. Nevertheless, hOB cultivated on BioOss® tend to demonstrate more beneficial levels of bone-specific markers. Sachar et al. emphasize the importance of three-dimensional cell embedding of hOB concerning the expression of cell-adhesion proteins and of other osteogenic marker genes [52]. Allografts obtained from older donors may be inferior to SBS in terms of their osteoconductive properties because of lower bone-mineral density. The SEM analysis revealed a greater amount of osteoblasts on SBS and a homogenously-distributed matrix deposition enabling three-dimensional embedding of the cells. Another explanation for the slightly inferior protein synthesis on ACB is the freeze-drying process and/or subsequent irradiation that might cause alterations in the anorganic structure. Organic or cellular remnants in the allograft might trigger immune reactions or impaired growth and secretion behavior of hOB despite the purging processes. Our hypothesis that in-vitro studies may predict in-vivo data could be confirmed; ACB and SBS had similar biocompatibility with osteoblasts and clinically equal applicability. However, considering the importance of clinically influencing factors, the informative and especially predictive value of the in-vitro studies seems to be limited.

Although we identified no significant group differences in the Alamar Blue® assay, a tendency towards a decrease of cell numbers and metabolic activity, especially in the ACB group, was seen. Despite, this demonstrates similar in-vitro biocompatibility of ACB and SBS. The decreasing cell number over time may be explained by the absent blood supply, which more efficiently transports nutrients and growth hormones compared to cell-culture-typical diffusion, a phenomenon described by e.g., Kneser et al. [66]. This would also explain the decline in osteogenic marker production that was especially seen in the ACB group after the first week.

The aim of this study was to compare and determine the clinical effectiveness of allogeneic cancellous bone grafts (ACB) and synthetic or highly-processed xenogeneic bone substitutes (SBS) to treat bony defects in different locations while considering epidemiological and custom-designed parameters. We hypothesized that there would be no difference between ACB and SBS in their effectiveness to regenerate and consolidate bone, a hypothesis we confirmed. Our study’s complication rate correlated highly significantly with the site of the bone defect, but not with the bone-graft material used.

We are aware of our study’s limitations. The SBS group contains a heterogenous amount of different xenogenic and synthetic bone-graft materials reflecting the clinical routine and occasional personal preferences. Nevertheless, the most important finding we made is the similar efficacy of ACB and SBS in the treatment of skeletal lesions independent of the graft material used. Other key factors such as the location of use and epidemiological aspects are much more likely to influence clinical outcomes than is the type of bone graft.

The main bone-grafting sites in our study were the humerus, tibia and foot in both intervention groups, in line with the usual complication rates in fracture care and implying that the occurrence of complications is not closely related to bone grafting. The consolidation rate, our other study endpoint, is primarily and significantly influenced by epidemiological factors, again not by the biomaterial applied. The in-vitro analysis comparing the different classes of bone substitutes is limited to biocompatibility assays in combination with measurements of factors defining an osteogenic environment. Although SEM supplemented this, the examination lacks other cell types as osteoclasts, osteocytes or chondrocytes that are necessary in-vivo for the natural bone turnover.

In allogeneic bone transplantation, the risk of microbiological contamination, transmission of viruses, delayed incorporation, and cellular and humoral immune reactions must be taken into consideration [67]. There have been many satisfactory clinical results reported following the application of allo- and xenografts [68, 69]. Nevertheless, Reikeras et al. describe complications after using deep-frozen allogeneic bone in 25 to 35 % of patients [70]. Sterilization and disinfection can reduce the immune response and the risk of infection, but they have major effects on the grafts’ mechanical and biological properties. Shegarfi et al. suspect that major histocompatibility complex peptide proteins from donor cells might survive the freezing process, making them subject to targeting by antigen-presenting cells after transplantation, thus triggering a long-term immune response [69]. Ghanaati et al. recently published data about organic and cellular remnants in allogenic and xenogenic bone blocks subjected to patented processing techniques that may cause immune reactions [11].

Due to the sterilization process prior to transplantation, allografts usually only provide osteoconductive properties, having lost their osteogenic capacity by purging from organic matrix and cell components. Nevertheless, our histologic findings confirm the existence of soft tissue as well as osteocytes, adipocytes and fibrocytes in the ACB graft. ACB’s high osteogenic capacity after the sterilization process seems doubtful. Lower levels of protein and hormone secretion on ACB in comparison to BioOss® may even reflect immune-reactions processes or compromised cell growth and cell metabolism due to effects on the mechanical and biological properties of ACB.

Individual differences such as age and the co-morbidities of our study’s osteoblast donors could have caused differences in growth characteristics, protein secretion levels and matrix deposition without attaining significance. Defects involving greater diameters and costs will probably present limitations regarding the use of SBS. The risks associated with microbiological contamination, virus transmission, and cellular or humoral immune reactions can only be reduced through continued research, in particular of xenogenic material.

## Conclusion

Both the materials we investigated (ACB vs. SBS) displayed similar in-vivo efficacy in the treatment of skeletal lesions. Other factors influencing the clinical outcome are the location of use and epidemiological parameters. Both materials were biocompatible in-vitro. We detected only marginal differences in the levels of protein and hormone secretion of hOBs cultivated on BioOss® or ACB.

## Availability of data and materials section

The datasets supporting the conclusions of this article are included within the article and its additional files.
