# Erratum to: Clinical trial and in-vitro study comparing the efficacy of treating bony lesions with allografts versus synthetic or highly-processed xenogeneic bone grafts

**DOI:** 10.1186/s12891-016-0976-0

**Published:** 2016-03-09

**Authors:** Eva Johanna Kubosch, Anke Bernstein, Laura Wolf, Tobias Fretwurst, Katja Nelson, Hagen Schmal

**Affiliations:** Department of Orthopedics and Trauma Surgery, Albert-Ludwigs University Medical Center, Freiburg, Germany; Department of Craniomaxillofacial Surgery, Albert-Ludwigs University Medical Center, Freiburg, Germany; Department of Orthopaedics and Traumatology, Odense University Hospital, Odense, Denmark; Department of Clinical Research, University of Southern Denmark, Odense, Denmark

## Erratum

After publication of the original article [1], the authors noticed an error in the ‘Methods’ section. Within the ‘Patients’ sub-section, the first item in the list of synthetic or highly processed xenogeneic bone substitutes (SBS) should have been given as Orthoss®, rather than BioOss®. The sentence should therefore have read as follows:

“The following SBS were used: Orthoss® (Geistlich, Wolhusen, Switzerland) in 41.4 %, and at clearly lower percentages, respectively: Norian SRS® (Synthes) (8.6 %), Chronos® (Synthes) (1.7 %), Atlantik® (Argomedical) (2.6 %), Alaska® (Argomedical) (0.9 %), Endobone® (Biomet) (4.3 %), Pyrost® (Stryker) (0.9 %), Nanostim® (Medtronic) (37.1 %), Actifuse® (Baxter) (0.9 %), Tutobone® (Novomedics) (0.9 %) and PerOssal® (Botiss) (0.9 %).”
